# Evidence for the Capability of Roxadustat (FG-4592), an Oral HIF Prolyl-Hydroxylase Inhibitor, to Perturb Membrane Ionic Currents: An Unidentified yet Important Action

**DOI:** 10.3390/ijms20236027

**Published:** 2019-11-29

**Authors:** Wei-Ting Chang, Yi-Ching Lo, Zi-Han Gao, Sheng-Nan Wu

**Affiliations:** 1 Division of Cardiovascular Medicine, Chi-Mei Medical Center, Tainan 71004 Taiwan; cmcvecho2@gmail.com; 2 Department of Biotechnology, Southern Taiwan University of Science and Technology, Tainan 71004, Taiwan; 3 Institute of Clinical Medicine, College of Medicine, National Cheng Kung University, Tainan 70101, Taiwan; 4 Department of Pharmacology, College of Medicine, Kaohsiung Medical University, Kaohsiung 80708, Taiwan; yichlo@kmu.edu.tw; 5 Department of Physiology, National Cheng Kung University Medical College, Tainan 70101, Taiwan; hhelen000111tw@gmail.com; 6 Institute of Basic Medical Sciences, National Cheng Kung University Medical College, Tainan 70101, Taiwan; 7 Department of Basic Medical Sciences, China Medical University Hospital, Taichung 40402, Taiwan

**Keywords:** roxadustat, delayed-rectifier K^+^ current, voltage-gated Na^+^ current, current kinetics, pituitary cell and heart cell

## Abstract

Roxadustat (FG-4592), an analog of 2-oxoglutarate, is an orally-administered, heterocyclic small molecule known to be an inhibitor of hypoxia inducible factor (HIF) prolyl hydroxylase. However, none of the studies have thus far thoroughly investigated its possible perturbations on membrane ion currents in endocrine or heart cells. In our studies, the whole-cell current recordings of the patch-clamp technique showed that the presence of roxadustat effectively and differentially suppressed the peak and late components of *I*_K(DR)_ amplitude in response to membrane depolarization in pituitary tumor (GH_3_) cells with an IC_50_ value of 5.71 and 1.32 μM, respectively. The current inactivation of *I*_K(DR)_ elicited by 10-sec membrane depolarization became raised in the presence of roxadustatt. When cells were exposed to either CoCl_2_ or deferoxamine (DFO), the *I*_K(DR)_ elicited by membrane depolarization was not modified; however, nonactin, a K^+^-selective ionophore, in continued presence of roxadustat, attenuated roxadustat-mediated inhibition of the amplitude. The steady-state inactivation of *I*_K(DR)_ could be constructed in the presence of roxadustat. Recovery of *I*_K(DR)_ block by roxadustat (3 and 10 μM) could be fitted by a single exponential with 382 and 523 msec, respectively. The roxadustat addition slightly suppressed *erg*-mediated K^+^ or hyperpolarization-activated cation currents. This drug also decreased the peak amplitude of voltage-gated Na^+^ current with a slowing in inactivation rate of the current. Likewise, in H9c2 heart-derived cells, the addition of roxadustat suppressed *I*_K(DR)_ amplitude in combination with the shortening in inactivation time course of the current. In high glucose-treated H9c2 cells, roxadustat-mediated inhibition of *I*_K(DR)_ remained unchanged. Collectively, despite its suppression of HIF prolyl hydroxylase, inhibitory actions of roxadustat on different types of ionic currents possibly in a non-genomic fashion might provide another yet unidentified mechanism through which cellular functions are seriously perturbed, if similar findings occur in vivo.

## 1. Introduction

The hypoxia-inducible factor (HIF) pathway alters gene expression in response to low oxygen tension. Under normal conditions, HIF-specific prolyl hydroxylases (HIF-PHs) initiate the degradation of oxygen-sensitive HIF isoforms. 

Roxadustat (FG-4592, 47 N-[(4-hydroxy-1-methyl-7-phenoxy-3-isoquinolinyl) carbonyl]-glycine, Ai Rui Zhuo^®^ in China), an analog of 2-oxoglutarate, is an orally administered, heterocyclic small molecule, known to inhibit HIF prolyl hydroxylases [1]. Of note, there are a growing number of studies to show that this drug could suppress HIF degradation and in this way induce erythropoietin expression, hence promoting erythropoiesis or preventing anemia in vivo [2,3,4,5,6,7,8,9,10,11].

Roxadustat has been also demonstrated to have therapeutic potential either for Parkinson’s diseases occurring in vitro or in vivo, for experimental retinal detachment, or for spinal cord injury, which are possibly through its regulation of redox reaction and mitochondrial function [5,12,13,14,15]. Alternatively, this compound could effectively exert neuroprotective effects against photoreceptor or neuronal cell death and prevent liver ischemia-reperfusion injury to donation after cardiac death [13,15,16]. The activity of K_V_ channels (e.g., eag1-mediated K^+^ channels) has been noted to be associated with oxygen homeostats and induction of angiogenesis in certain types of tumors [17]. Of particular interest, the HIF-1α expression in pituitary gland including pituitary tumor (GH_3_) cells has shown that it is closely linked to the occurrence or progression of pituitary adenoma [16,18,19,20,21,22,23,24].

On the other hand, roxadustat was reported to possess some unexpected side effects [25]. For example, a recent study demonstrated that inhibitors of HIF prolyl hydroxylases could effectively exert neuroprotective actions which are thought to be independent of its inhibitory effects on HIF prolyl hydroxlyases [26]. Roxadustat was also found to exert direct effects on skeletal myotube [27]. However, of note, whether roxadustat is capable of producing any perturbations on the level of surface membrane including on ionic channels in different types of cells is not thoroughly investigated, despite its clinical approval for anemia in chronic kidney disease with or without the dialysis [1,4,5,8,9,10]. 

Because of the aforementioned considerations, rising attempts were made to evaluate whether roxadustat or other related compounds could produce any modifications on various types of ionic currents (e.g., delayed-rectifier K^+^ current [*I*_K__(DR__)_], *erg*-mediated K^+^ current [*I*_K(erg)_], and voltage-gated Na^+^ current [*I*_Na_]) in pituitary tumor (GH_3_) cells. Moreover, earlier numerous studies have reported the effective regulations of HIF-1α expression present in heart-derived H9c2 myoblasts [24,28,29,30,31,32,33,34,35]. Thus, we further decided to investigate the possible actions of roxadustat on *I*_K(DR)_ in these cells. In the present study, we provide the evidence to unravel that roxadustat is able to modify membrane ionic currents in pituitary tumor (GH_3_) cells and in cardiac H9c2 cells, those actions of which are conceivably of clinical or pharmacological relevance and tend to be upstream of its inhibitory action on HIF prolyl hydroxylases.

## 2. Results

### 2.1. Effect of Roxadustat on Delayed-Rectifier K^+^ Current (I_K(DR)_) Recorded from Pituitary Tumor (GH_3_) Cells

**Figure 1:**
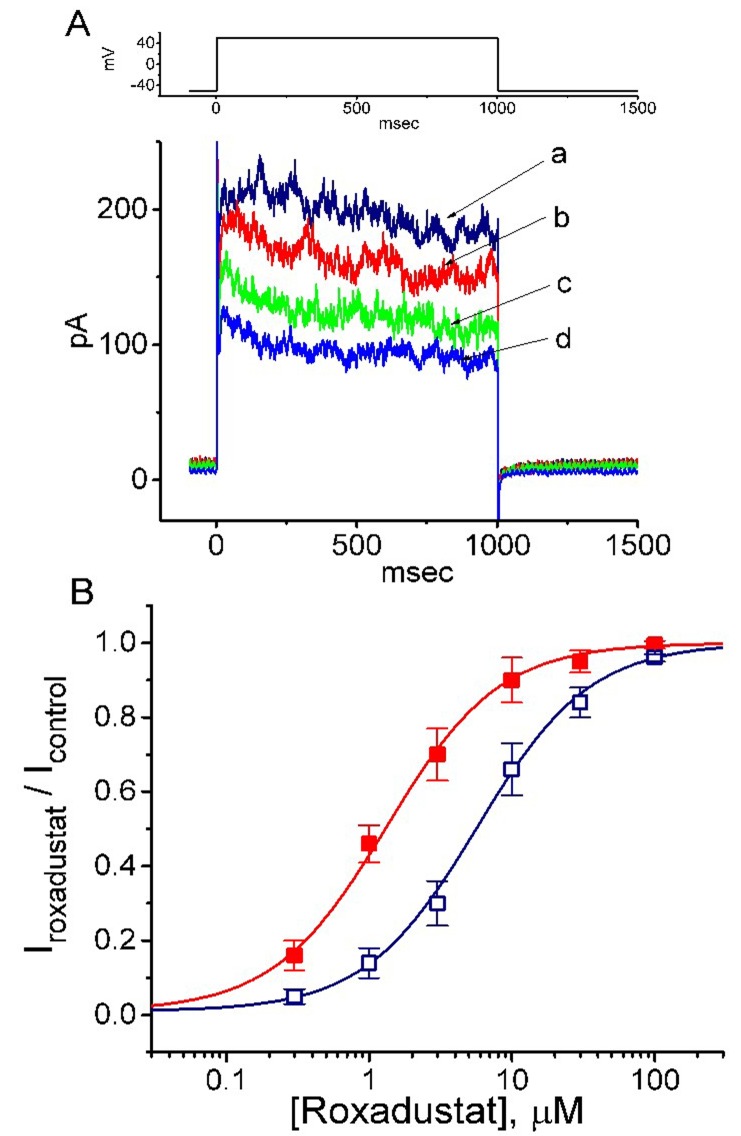
Inhibitory effect of roxadustat on delayed-rectifier K^+^ current (*I*_K(DR)_) in pituitary GH_3_ cells. In these whole-cell current recordings, cells were bathed in Ca^2+^-free Tyrode’s solution containing 1 μM tetrodotoxin and 5 mM CdCl_2_, and the recording pipette was backfilled with a K^+^-containing solution. (**A**) Representative *I*_K(DR)_ traces in responses to membrane depolarization (as indicated in the upper part). a: control, b: 0.3 μM roxadustat; c: 1 μM roxadustat; d: 3 μM roxadustat. (**B**) Concentration-dependent effects of roxadustat on *I*_K(DR)_. The *I*_K(DR)_ was evoked by membrane depolarization from −50 to +50 mV. Current amplitudes obtained during cell exposure to different concentrations (0.3–30 μM) of roxadustat were measured at the beginning (□, peak *I*_K(DR)_) and end (■, late *I*_K(DR)_) of depolarizing pulses. Each point indicates the mean ± standard error of the mean (SEM, *n* = 8–11). The values of IC_50_ and n_H_ for roxadustat-mediated inhibition of late *I*_K(DR)_ were estimated to be 1.32 μM and 1.1, respectively, and those for peak *I*_K(DR)_ were 5.71 μM and 1.1, respectively. Of note, the roxadustat addition is able to differently suppress the amplitude of peak and late *I*_K(DR)_ in GH_3_ cells, with no appreciable change in the Hill coefficient of the curve.

In the first stage of experiments, we examined whether this drug has any perturbation on *I*_K(DR)_ inherently in these cells. In order to avoid the contamination of Ca^2+^-activated K^+^ currents [36,37], we bathed cells in Ca^2+^-free, Tyrode’s solution, and during the measurements, we filled the recording electrode with a K^+^-containing solution. The composition of these solutions is detailed above. As the whole-cell configuration was firmly established, we maintained the examined cell at −50 mV and the depolarizing voltage step to +50 mV with a duration of 1 sec was then applied to evoke *I*_K(DR)_. As shown in Figure 1A, within 1 min of exposing the cell to different roxadustat concentrations, the amplitude of *I*_K(DR)_ was progressively decreased; moreover, the decaying time course of the current in response to maintained depolarization became faster in their presence. The concentration-dependent relationships of roxadustat effect on *I*_K(DR)_ amplitude measured at the beginning (i.e., peak current) or end (i.e., late current) of the depolarizing pulse were constructed and are hence plotted in Figure 1B. The IC_50_ values required for roxadustat-mediated suppression of peak and late *I*_K(DR)_ were calculated to be 5.71 and 1.32 μM, respectively; however, no measurable change in Hill coefficient of the relationship was found. Therefore, due primarily to the increase of inactivation time course in response to membrane depolarization, the half maximal inhibitory concentration (IC50) value for roxadustat was conceivably noted to be fivefold less in *I*_K(DR)_ amplitude measured at the beginning of depolarizing step than in that at the end of pulse.

### 2.2. Effect of Roxadustat on the Inactivation Process of I_K(DR)_ Elicited by 10-sec Membrane Depolarization

**Figure 2:**
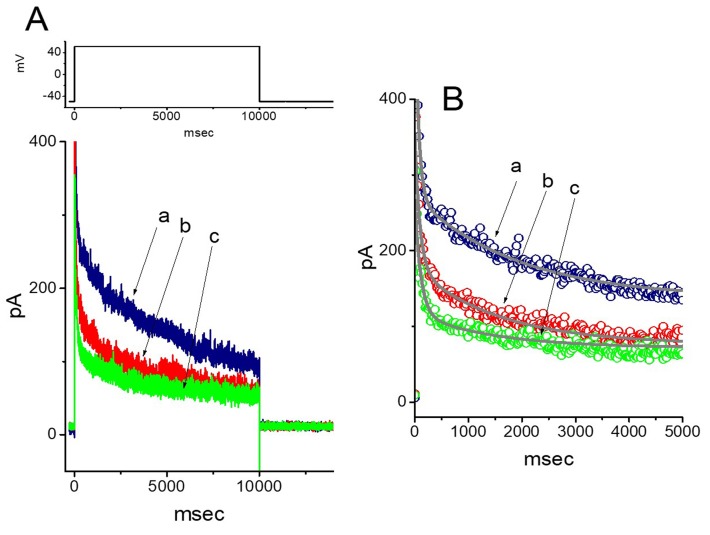
Effect of roxadustat on *I*_K(DR)_ elicited by long-lasting membrane depolarization in GH_3_ cells. In this set of experiments, the duration of clamp pulse from −50 to +50 mV was maintained for 10 sec, and the tested cells were depolarized from −50 to +50 mV. (**A**) Representative *I*_K(DR)_ traces obtained in the control (a), and during the exposure to 1 μM roxadustat (b) and 3 μM roxadustat (c). The upper part denotes the voltage protocol applied. (**B**) Continuous line in each *I*_K(DR)_ trajectory corresponds to the least-squares fit to two-exponential function. The data points in each trace were skipped by 20 for clear representation. a: control; b: 1 μM roxadustat; c: 3 μM roxadustat. Of note, the presence of roxadustat, in addition to the reduction of *I*_K(DR)_ amplitude, decreases the τ_inact(F)_ and τ_inact(S)_ values of *I*_K(DR)_ in response to 10-sec membrane depolarization.

In order to determine the inhibitory effect of roxadustat on the inactivation of *I*_K(DR)_, a 10 sec long and sustained depolarization from −50 to +50 mV was applied to the cell. As shown in Figure 2, upon 10-sec membrane depolarization, the trajectory of *I*_K(DR)_ inactivation in the control became slowly decayed with the values of fast (τ_inact(F)_) and slow component (τ_inact(S)_) of current inactivation of 67 ± 9 and 1908 ± 102 msec (*n* = 9), respectively. Importantly, as the cells were exposed to roxadusat, the inactivation rate of *I*_K(DR)_ was escalated and the values of τ_inact(F)_ and τ_inact(S)_ were hence shortened, as evidenced by a significant reduction of τ_inact(F)_ and τ_inact(S)_ values to 54 ± 8 and 1442 ± 78 msec (*n* = 9, *p* < 0.05), respectively, during the exposure to 3 μM roxadustat. After washout of the agent, the values of τ_inact(F)_ and τ_inact(S)_ returned to 64 ± 7 and 1894 ± 98 msec (*n* = 7), respectively; moreover, current amplitude returned to the control level. Therefore, roxadustat, in addition to the suppression of current amplitude, was capable of decreasing τ_inact(F)_ and τ_inact(S)_ values of the current elicited by such long-lasting membrane depolarization.

### 2.3. Comparisons among Effects of Roxadustat, CoCl_2_ and Deferoxamine on I_K(DR)_ Amplitude in GH_3_ Cells

**Figure 3:**
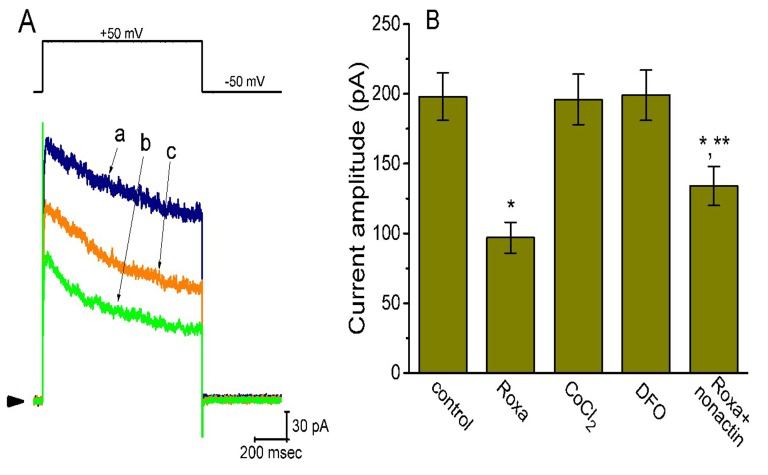
Comparisons between effects of roxadustat (3 μM), CoCl_2_ (1 mM), deferoxamine (30 μM), and roxadustat (3 μM) plus nonactin (10 μM) on *I*_K(DR)_ amplitude in GH_3_ cells. In these experiments, we bathed cells in Ca^2+^-free, Tyrode’s solution and the pipette was filled with K^+^-containing solution. (**A**) Superimposed *I*_K(DR)_ obtained in the control (a) and during the exposure to 3 μM roxadustat (b) and 3 μM roxadustat plus 10 μM nonactin (c). The upper part indicates the voltage protocol used and arrowhead in the left side of panel is the zero current level. (**B**) Summary bar graph showing the effects of roxadustat, CoCl_2_, deferoxamine, and roxadustat plus nonactin on *I*_K(DR)_ amplitude. The current amplitude at the end of depolarizing command from −50 to +50 mV was taken. Each bar indicates the mean ± SEM (*n* = 6–9 for each bar). * Significantly different from control (*p* < 0.05) and ** significantly different from roxadustat alone group (*p* < 0.05). Roxa: 3 μM roxadustat; CoCl_2_: 1 mM CoCl_2_; DFO: 30 μM deferoxamine; nonactin: 10 μM nonactin.

Previous studies have demonstrated that CoCl_2_ and deferoxamine (DFO, an iron chelator) are hypoxia mimetic reagents. DFO can suppress the activity of iron-dependent prolyl hydroxylase, while CoCl_2_ activates iron-independent induction of HIF-1α [38]. However, as shown in Figure 3, the addition of CoCl_2_ or DFO to bath could not modify the *I*_K(DR)_ elicited in response to membrane depolarization. However, in continued presence of roxadustat (3 μM), subsequent addition of nonactin (10 μM), a K^+^-selective ionophore, significantly attenuated roxadustat-mediated suppression of *I*_K(D__R)_. As a result, the results led us to reflect that roxadustat-mediated inhibition of *I*_K(DR)_ in GH_3_ cells tends to be not attributable to its effect on the accumulation of HIF-1α.

### 2.4. Effect of Roxadustat on the Steady-State Inactivation Curve of I_K(DR)_ Recorded from GH_3_ Cells

**Figure 4:**
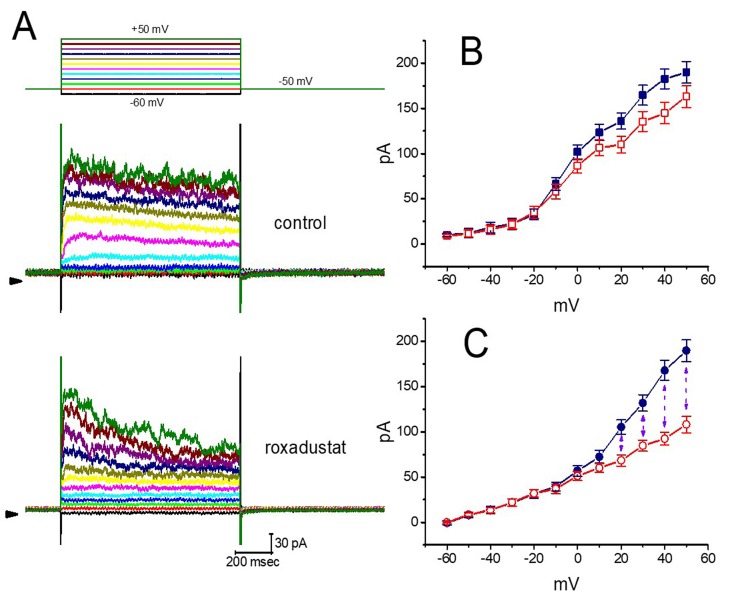
Averaged current–voltage (*I–V*) relationships of *I*_K(DR)_ taken with or without roxadustat addition. In these experiments, the examined cells were maintained at −50 mV and a series of voltage pulses from −60 to +50 mV in 10-mV increments were applied at a rate of 0.1 Hz. (**A**) Representative *I*_K(DR)_ traces obtained in the absence (upper) and presence (lower) of 3 μM roxadustat. The upper part indicates the voltage protocol applied and the arrowhead shown in the left inside each panel is the zero current level. Calibration mark in the right lower corner applies to all panels. (**B**) Averaged *I–V* relationships of *I*_K(DR)_ in the control (i.e., in the absence of roxadustat). Current amplitudes at different levels of voltage were measured at the beginning (■) and end (□) of voltage steps. (**C**) Averaged *I–V* relationships of *I*_K(DR)_ in the presence of 3 μM roxadustat. Current amplitudes at different levels of command steps were measured at the beginning (●) and end (○) of voltage steps. Each point shown in (B) and (C) indicates the mean ± SEM (*n* = 9). The vertical dashed line shown in (C) indicates considerable difference between peak and late *I*_K(DR)_ as cells were exposed to 3 μM roxadustat.

**Figure 5:**
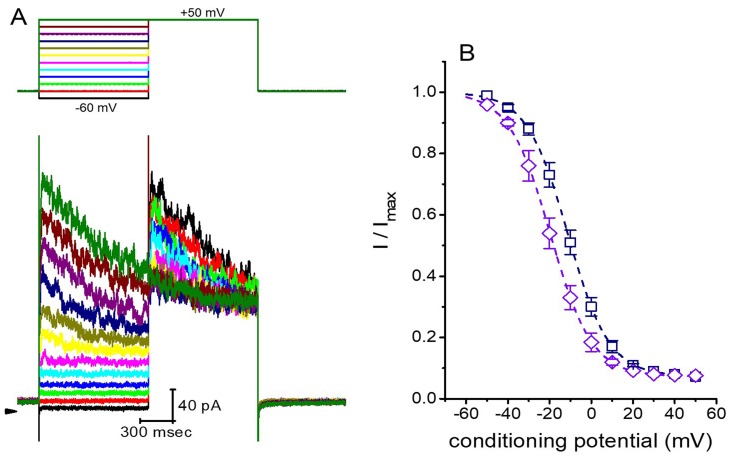
Effect of roxadustat on the steady-state inactivation curve of *I*_K(DR)_ in GH_3_ cells. These experiments were conducted in a two-step voltage clamp protocol. (**A**) Representative *I*_K(DR)_ traces obtained in the presence of 3 μM roxadustat. The upper part denotes the voltage protocol used, and arrowhead in the left side inside panel (A) is zero current level. Calibration mark in the right lower corner applies to all current traces. (**B**) Steady-state inactivation curve of *I*_K(DR)_ obtained in the presence of 3 μM (□) and 10 μM roxadustat (◇). Each point indicates the mean ± SEM (*n* = 8). The smooth curves were least-squares fitted by a Boltzmann function (detailed under Materials and Methods).

The steady-state inactivation curve of *I*_K(DR)_ in the presence of roxadustat was further studied. In these experiments, cells were immersed in Ca^2+^-free Tyrode’s solution and the recording electrode was filled with K^+^-containing solution (Figure 4). As whole-cell configuration was achieved, we applied a two-pulse protocol (i.e., through analog-to-digital conversion) to the examined cells which were exposed to different roxadustat concentrations. The inactivation parameters of *I*_K(DR)_ were appropriately measured in the presence of 3 or 10 μM roxadustat. As depicted in Figure 5, the normalized amplitudes of *I*_K(DR)_ (i.e., I/I_max_) were constructed against the conditioning potential and the continuous curve was fitted with the goodness of fit by a modified Boltzmann function described under Materials and Methods. In the presence of 3 μM roxadustat, *V*_1/2_ = −11.2 ± 1.2 mV, *q* = 2.6 ± 0.2 *e* and *a* = 0.074 ± 0.002 (*n* = 8), while in the presence of 10 μM roxadustat, *V*_1/2_ = -19.4 ± 1.4 mV, *q* = 2.5 ± 0.2 *e* and *a* = 0.074 ± 0.002 (*n* = 8). Results from these experiments showed that during cell exposure to different roxadustat concentrations, the inactivation parameter (i.e., V_1/2_ value) of *I*_K(DR)_ measured from GH_3_ cells could be altered, despite no appreciable change in the gating charge (*q*) of the current.

### 2.5. Effect of Roxadustat on the Recovery of I_K(DR)_ Block in GH_3_ Cells

**Figure 6:**
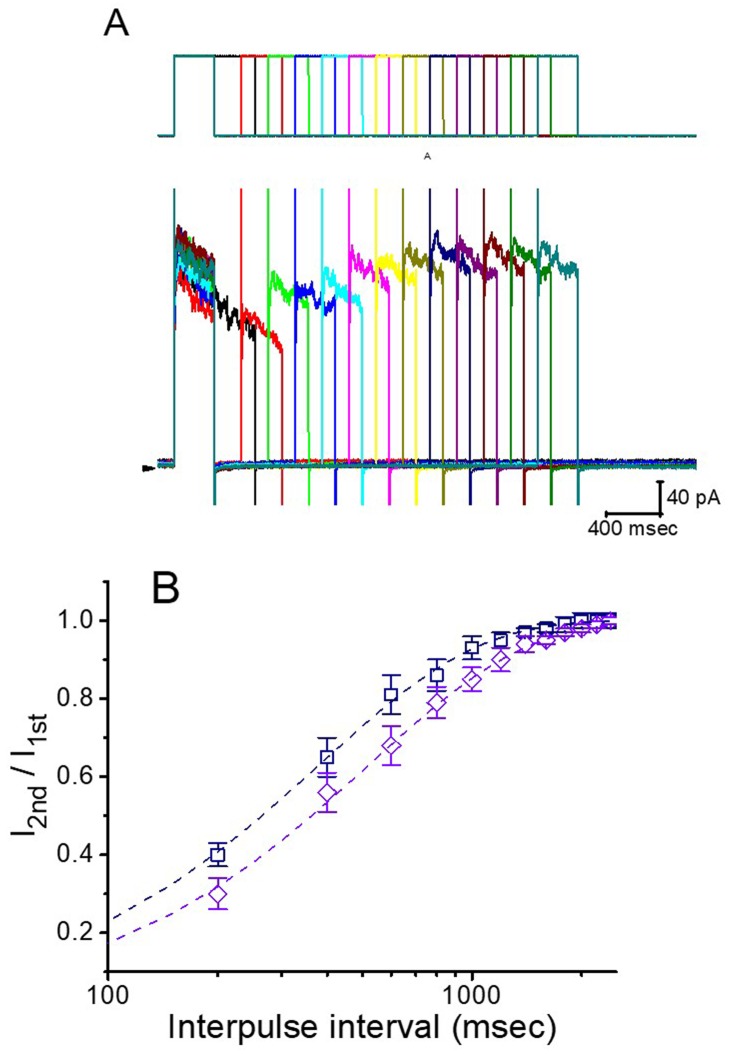
Time course of the recovery of *I*_K(DR)_ block in the presence of roxadustat. In these experiments, another set of two-pulse voltage protocol was applied to the examined cells. (**A**) Superimposed *I*_K(DR)_ trace in response to two-pulse voltage protocol with different interpulse durations (indicated in the upper part), as the cell was exposed to 3 μM roxadustat. (**B**) Time course of recovery from *I*_K(DR)_ inactivation obtained in the presence of 3 μM roxadustat (□) and 10 μM roxadustat (◇) (mean ± SEM; *n* = 8 for each point). Smooth dashed lines were fitted by single exponential. Note that abscissa (i.e., interpulse interval) in the graph is illustrated at logarithmic scale.

In order to evaluate roxadustat-induced block of *I*_K(DR)_ in these cells, we further investigated the recovery from current inactivation caused by this agent. In this series of experiments, a stimulation protocol consisting of a first (conditioning) depolarizing pulse which is adequately long to allow block to reach a steady state was used. While cells were exposed to roxadustat (3 or 10 μM), the *I*_K(DR)_ traces were elicited by the following voltage protocol; that is, the membrane potential was stepped to +50 mV from −50 mV for a variable time, after that a second depolarizing pulse (test pulse) was applied at the same potential as the coditioning pulse (Figure 6). Then, the ratios of the peak *I*_K(DR)_, in response to the second (test, Ipost) and first (conditioning, Ipre) pulse, were taken as a measure of recovery from *I*_K(DR)_ block and are plotted versus interpulse interval. As depicted in Figure 6, the *I*_K(DR)_ recovery from block during the exposure to 3 or 10 μM roxadustat was constructed and the time courses could be described by single exponential with the time constants of 382 ± 16 and 523 ± 22 msec (*n* = 8). It is likely, from the present results, that the recovery of *I*_K(DR)_ block produced by the presence of roxadustat in GH_3_ cells was prolonged as its concentration was increased, and that the delayed recovery caused by this agent may result largely from open channel block, as reported previously for aconitine-induced block of *I*_K(DR)_ [39].

### 2.6. Effect of Roxadustat on Erg-Mediated K^+^ Current (I_K(erg)_) in GH_3_ Cells

**Figure 7:**
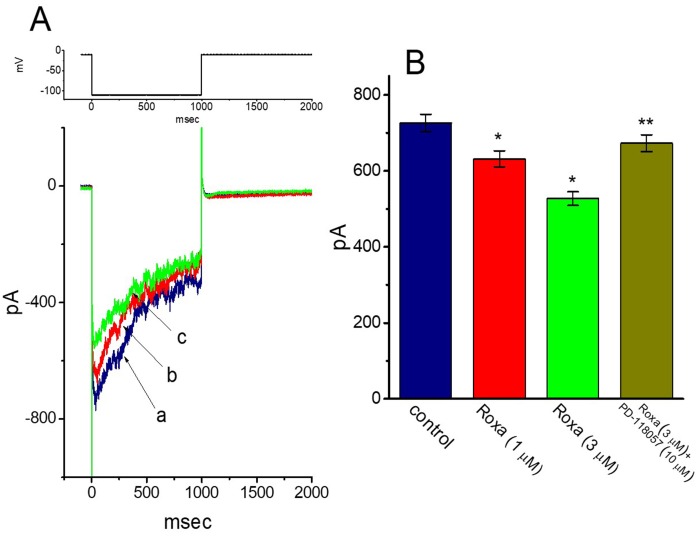
Effect of roxadustat on *erg*-mediated K^+^ current (*I*_K(erg)_) in GH_3_ cells. In this set of experiments, cells were bathed in high-K^+^, Ca^2+^-free solution and the recording pipette was filled with K^+^-containing solution. (**A**) Representative *I*_K(erg)_ traces obtained in the absence (a) and presence of 1 μM roxadustat (b) or 3 μM roxadustat (c). The upper part is the voltage protocol used for elicitation of deactivating *I*_K(erg)_. (**B**) Summary bar graph showing the effects of roxadustat (Roxa) and roxadustat plus PD-118057 on *I*_K(erg)_ amplitude in GH_3_ cells. To evoke deactivating *I*_K(erg)_, each cell was maintained at -10 mV and the hyperpolarizing pulse to -110 mV with a duration of 1 sec was applied. *I*_K(erg)_ amplitude was measured at the beginning of hyperpolarizing pulse. Each bar indicates the mean ± SEM (*n* = 8 for each bar). * Significantly different from control (*p* < 0.05) and ** significantly different from 3 μM roxadustat alone group.

The *I*_K(erg)_ has been previously reported to be linked to the regulation of HIF (17). We next tested whether roxadustat can modify another type of K^+^ current (i.e., *I*_K(erg)_) which is inherently present in GH_3_ cells [40,41,42]. These experiments were conducted in cells bathed in high-K^+^, Ca^2+^-free solution, and we filled the recording pipette with K^+^-containing solution. As shown in Figure 7, the deactivating *I*_K(erg)_ could be readily evoked by membrane hyperpolarization to −110 mV [40]. The addition of 1 or 3 μM roxadustat decreased the peak amplitude of *I*_K(erg)_ to 632 ± 21 pA (*n* = 8, *p* < 0.05) or 527 ± 18 pA (*n* = 8, *p* < 0.05), respectively, from a control value of 726 ± 23 pA (*n* = 8). In the continued presence of 3 μM roxadustat, further addition of 10 μM PD-118057 notably reversed *I*_K(erg)_ amplitude to 673 ± 22 (*n* = 8, *p* < 0.05). PD-118057 was previously shown to activate *I*_K(erg)_ [43]. Therefore, as compared with its suppressive effect on *I*_K(DR)_ described above, the *I*_K(erg)_ in GH_3_ cells is relatively resistant to modification by roxadustat.

### 2.7. Suppressive Effect of Roxadustat on Hyperpolarization-Activated Cation Current (I_h_) Recorded from GH_3_ Cells

**Figure 8:**
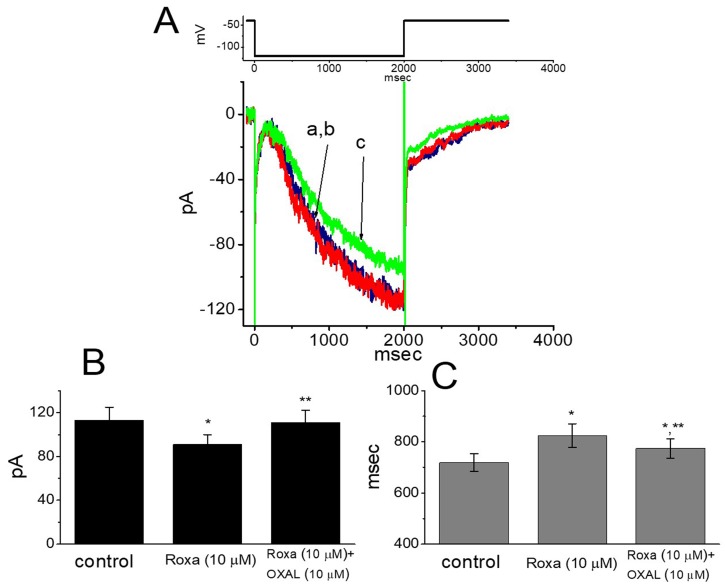
Effect of roxadustat on hyperpolarization-activated cation current (*I*_h_) in GH_3_ cells. These experiments were conducted in cells bathed in Ca^2+^-free Tyrode’s solution and the pipette was filled with K^+^-containing solution. (**A**) Representative *I*_h_ traces obtained in the absence (a) and presence of 3 μM roxadustat (b) or 10 μM roxadustat (c). The upper part is the voltage protocol used. Panels (**B**) and (**C**), respectively, indicate the effect of roxadustat and roxadustat plus oxaliplatin (OXAL) on the amplitude and the activation time constant of *I*_h_ elicited by membrane hyperpolarization from −40 to −120 mV with a duration of 2 s. Each bar indicates the mean ± SEM (*n* = 8 for each bar). * Significantly different from controls (*p* < 0.05) and ** significantly different from 10 μM roxadustat alone group (*p* < 0.05).

Whether roxadustat can modify the amplitude and gating of *I*_h_ in these cells was further tested in this study. In these experiments, cells were bathed in Ca^2+^-free, Tyrode’s solution and we filled the patch electrode with K^+^-containing solution. As the examined cell was maintained at −40 mV, a 2-sec membrane hyperpolarization to −120 mV readily evoked the *I*_h_, the property of which, in accordance with previous observations [44], displayed a considerably slow activation (i.e., a slowly activating inward [depolarizing] current). Moreover, as cells were exposed to 3 μM roxadustat, no significant change in the *I*_h_ amplitude by long-lasting hyperpolarizing command was demonstrated. However, as depicted in Figure 8, at 10 μM roxadustat decreased the *I*_h_ amplitude from 113 ± 12 to 91 ± 9 pA (*n* = 8, *p* < 0.05). Subsequent application of 10 μM oxaliplatin, still in the presence of 10 μM roxadustat, could significantly reverse *I*_h_ amplitude to 111 ± 11 pA (*n* = 8, *p* < 0.05). Meanwhile, the addition of 10 μM roxadustat raised the activation time constant of *I*_h_ from 719 ± 34 to 824 ± 46 msec (*n* = 8, *p* < 0.05), and subsequent addition of 10 μM oxaliplatin reduced the time constant of current activation to 774 ± 38 msec (*n* = 8, *p* < 0.05). Oxaliplatin was recently noted to increase the amplitude of *I*_h_ in response to long-lasting membrane hyperpolarization [25]. Therefore, roxadustat at a concentration of 10 μM can decrease *I*_h_ amplitude as well as slow the activation time course of the current.

### 2.8. Inhibitory Effect of Roxadustat on Voltage-Gated Na^+^ Current (I_Na_) in GH_3_ Cells

**Figure 9:**
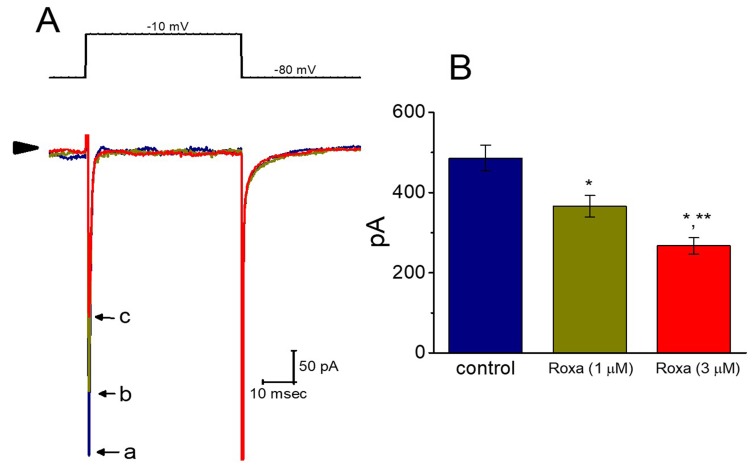
Effect of roxadustat on voltage-gated Na^+^ current (*I*_Na_) in GH_3_ cells. In these whole-cell recording experiments, we bathed cells in Ca^2+^-free Tyrode’s solution and, during the measurements, we filled the pipette with Cs^+^-containing solution, the composition of which is described under Materials and Methods. (**A**) Representative *I*_Na_ traces taken in the control (a) and during cell exposure to 1 μM roxadustat (b) or 3 μM roxadustat (c). The upper part of (**A**) is the voltage protocol (i.e., the examined cell was rapidly depolarized from −80 mV to −10 mV) and arrowhead denotes the zero current level. (**B**) Summary bar graph showing the inhibitory effect of roxadustat on peak *I*_Na_ elicited by rapid membrane depolarization from −80 to −10 mV. Current amplitude was measured at the beginning of rapid depolarization. Each bar represents the mean ± SEM (*n* = 9). * Significantly different from control (*p* < 0.05) and ** significantly different from 1 μM roxadustat group (*p* < 0.05). Note that the presence of roxadustat, in addition to the decreased amplitude of peak *I*_Na_, slowed the inactivation time course of the current.

We also ascertained whether the *I*_Na_ present in GH_3_ cells was subject to any perturbations by roxadustat. In these experiments, cells were bathed in Ca^2+^-free, Tyrode’s solution, and the recording pipettes used were filled with Cs^+^-containing solution, the composition of which is detailed under Materials and Methods. As shown in Figure 9, the addition of roxadustat produced a progressive reduction of peak *I*_Na_ in these cells. For example, within 1 min of exposing cells to 1 or 3 μM roxadustat, the peak amplitude of *I*_Na_ in response to rapid membrane depolarization was significantly decreased to 366 ± 27 pA (*n* = 8, *p* < 0.05) or 267 ± 21 pA (*n* = 8, *p* < 0.05), respectively, from a control value of 486 ± 32 pA (*n* = 8). After washout of this compound, peak *I*_Na_ was returned to 359 ± 25 pA (*n* = 8, *p* < 0.05). 

However, it needs to be mentioned that, as described previously in the telmisartan actions on *I*_Na_ [41], the inactivation rate of *I*_Na_ in response to brief sustained depolarization became slowed in the presence of roxadustat. As cells were exposed to 1 or 3 μM roxadustat, the inactivation time constant of *I*_Na_ was increased to 1.31 ± 0.21 msec (*n* = 8, *p* < 0.05) or 1.63 ± 0.35 msec (*n* = 9, *p* < 0.05), respectively, from a control value of 0.84 ± 0.17 msec (*n* = 8). Therefore, distinguishable from roxadustat-induced increase of *I*_K(DR)_ inactivation, the inactivation rate of peak *I*_Na_ elicited by brief depolarization was apparently depressed in the presence of roxadustat. This occurrence indicates that roxadustat can decrease peak *I*_Na_ amplitude concomitantly with the attenuation of current inactivation rate in GH_3_ cells.

### 2.9. Effect of Roxadustat on I_K(DR)_ Recorded from Heart-Derived H9c2 Cells

**Figure 10:**
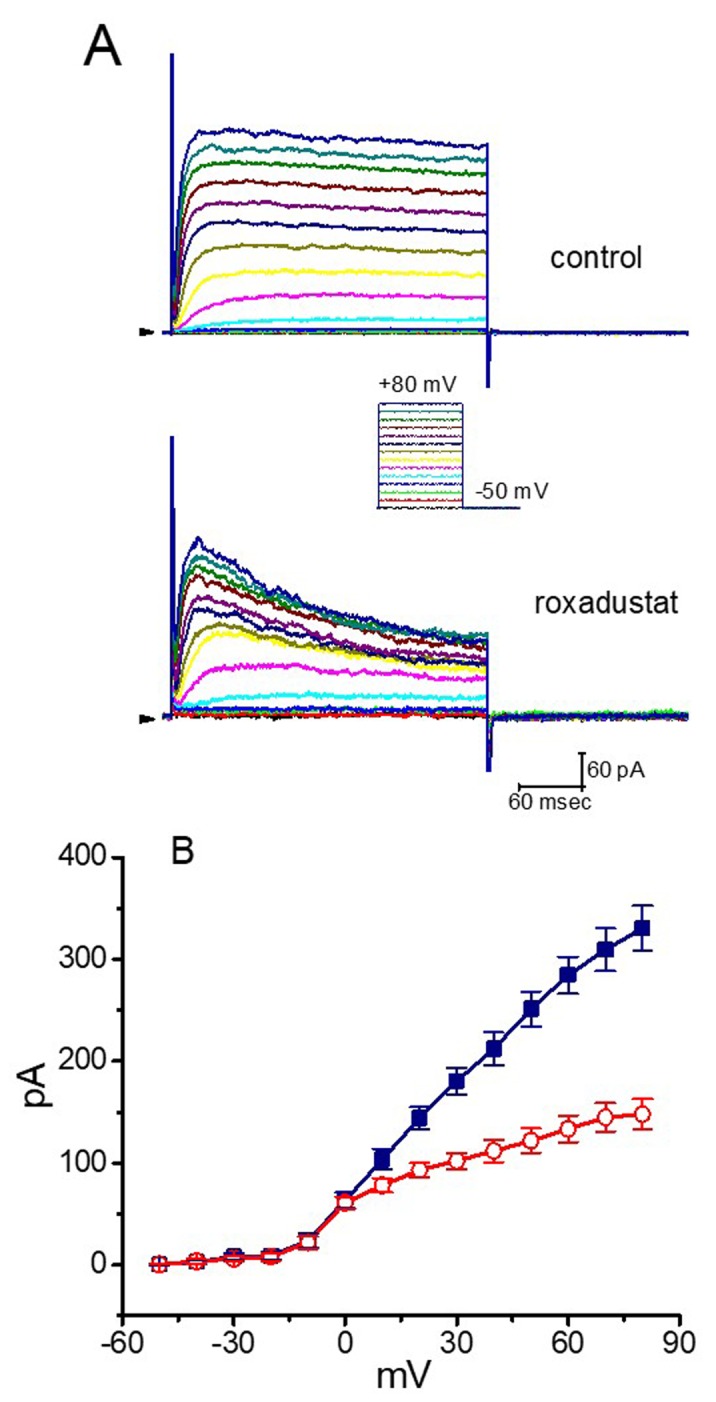
Effect of roxadustat on *I*_K(DR)_ recorded from heart-derived H9c2 cells. Experimental protocol used is similar to that for measurement of *I*_K(DR)_ in GH_3_ cells. (**A**) Representative *I*_K(DR)_ traces obtained in the absence (upper) and presence (lower) of 3 μM roxadustat. Inset indicates the voltage protocol applied. Arrowhead in the left side of each panel marks the zero current level, while calibration mark in the right lower corner applies to all current traces. (**B**) Averaged *I–V* relationships of *I*_K(DR)_ with or without addition of 3 μM roxadustat (mean ± SEM; *n* = 8 for each point). Current amplitude was measured at the end of 300-msec depolarizing pulse. ■: control; □: in the presence of 3 μM roxadustat.

A number of investigations have noted that the expression of HIF-1α in heart-derived H9c2 myoblasts could be delicately regulated [24,28,29,30,31,35]. In another set of experiments, we thus investigated the possible perturbations of roxadustat on *I*_K(DR)_ inherently in H9c2 cells. As illustrated in Figure 10, the *I*_K(DR)_ in response to membrane depolarization from −50 mV to a series of voltage steps as described previously [45,46] could be readily evoked with an outwardly rectifying property. Importantly, the presence of 3 μM roxadustat was found to suppress the *I*_K(DR)_ amplitude effectively elicited throughout the entire voltage-clamp steps in these cells. For example, at the level of +60 mV, the addition of 3 μM roxadustat diminished current amplitude (i.e., late *I*_K(DR)_) by 53 ± 3 % from 284 ±18 to 133 ± 13 pA (*n* = 8, *p* < 0.05). Similar to the previous results shown in GH_3_ cells, the presence of roxadustat can significantly but differentially suppress the peak and late amplitude of *I*_K(DR)_ in cardiac H9c2 cells.

### 2.10. Effect of Roxadustat on I_K(DR)_ in High Glucose-Treated H9c2 Cells

**Figure 11:**
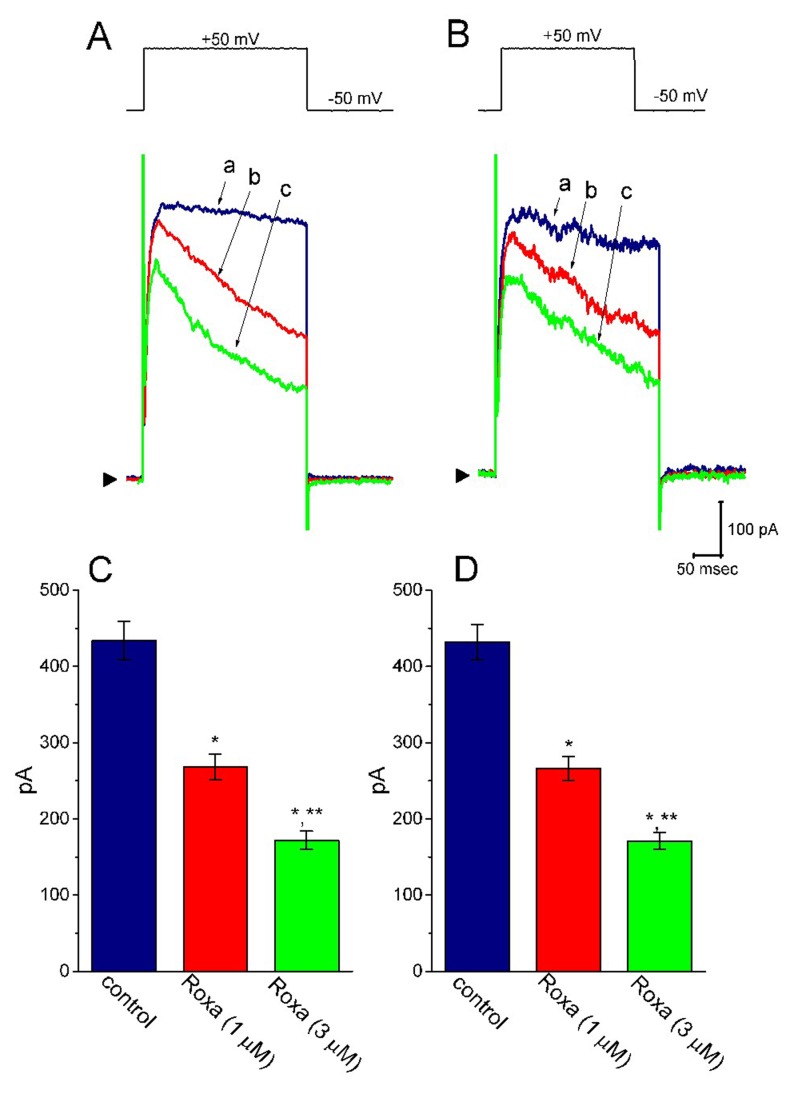
Effect of roxadustat on *I*_K(DR)_ in high glucose-treated H9c2 cells. In these experiments, H9c2 cells were incubated in normal glucose (5.5 mM) or high glucose (30 mM) medium for 24 h. In (**A**) and **(B),** superimposed *I*_K(DR)_ traces obtained in the control (a) and during the exposure to 1 μM roxadustat (b) and 3 μM roxadustat (c) were taken in the cell treated with normal glucose (5.5 mM) and high glucose (30 mM), respectively. The upper part in each panel denotes the voltage protocol, the calibration mark in the lower part applies all current trace, and arrowhead at the left side of currents in each panel is the zero current level. In (**C**,**D**), summary bar graphs illustrate the effects of roxadustat on the *I*_K(DR)_ amplitude recorded from H9c2 cells treated with normal glucose (5.5 mM) and high glucose (30 mM), respectively (mean ± SEM; *n* = 7 for each bar). * Significantly different from control (*p* < 0.05) and ** significantly different from 1 μM roxadustat alone group (*p* < 0.05).

Previous studies have demonstrated that roxadustat could alleviate high glucose-induced cell injury [30,47]. The challenging of cells with high glucose has been also shown to suppress the activity of HIF prolyl hydroxylase along with the upregulation of HIF-1α [30,48]. For these reasons, we further studied whether in high glucose-treated H9c2 cells, the biophysical properties of *I*_K(DR)_ are able to be altered and whether the roxadustat addition could still have any modifications on *I*_K(DR)_ in these cells. As shown in Figure 11, the *I*_K(DR)_ in response to membrane depolarization in these cells (i.e., high glucose-treated cells) was quite indistinguishable from that in control cells (i.e., little current inactivation in response to maintained depolarization). Furthermore, within 1 min of exposing high glucose-treated cells to roxadustat, the peak and late amplitudes of *I*_K(DR)_ were differentially suppressed. For example, roxadustat (3 μM) decreased the late *I*_K(DR)_ (i.e., current amplitude measured at the end of depolarizing step) from 432 ± 23 to 171 ± 11 (*n* = 7, *p* < 0.05) in H9c2 cells treated with high glucose (30 mM). Therefore, the presence of roxadustat remains efficacious at modifying the amplitude and gating of *I*_K(DR)_ in high glucose-treated cells.

## 3. Discussion

Our results demonstrated that in GH_3_ cells, the presence of roxadustat (FG-4592) differentially produced an inhibitory action on the peak and late component of *I*_K(DR)_ in a concentration-dependent manner. The values of τ_inact(F)_ and τ_inact(S)_ of *I*_K(DR)_ elicited by 10-sec membrane depolarization were reduced. The steady-state inactivation curve of *I*_K(DR)_ as well as the recovery of *I*_K(DR)_ block produced by the presence of roxadustat was clearly demonstrated in this study. However, when cells were exposed to either CoCl_2_ or DFO, the *I*_K(DR)_ elicited by membrane depolarization was not modified. This agent slightly suppressed the amplitude of *I*_K(erg)_ and *I*_h_. It also inhibited *I*_Na_ with a clear slowing in the inactivation rate of the current. The modifications of ionic currents by roxadustat presented herein tend to be acute in onset, and they could be independent of its interaction with HIF prolyl hydroxylases, shortly before the molecules pass through surface membrane. The action of roxadustat on the amplitude and gating *I*_K(DR)_ is thought to be through its preferential binding to the open-inactivated state(s) of the K_V_ channel. 

Roxadustat was previously reported to disrupt mitochondrial oxygen consumption present in skeletal C2C12 myotube [27]. Earlier studies have demonstrated the presence of ATP-sensitive K^+^ channels functionally expressed in GH_3_ and H9c2 cells [41,49,50]. It has been also shown that mitochondrial function found in different types of neurons may be stabilized by the presence of roxadustat [12,13,15,51]. However, it is important to note that in our whole-cell current recordings, the pipette solution contained 3 mM ATP, a value that is adequate to suppress the activation of ATP-sensitive K^+^ (K_ATP_) channels. Furthermore, subsequent addition of diazoxide (10 μM), an activator of K_ATP_ channels, but still in the presence of roxadustat, was unable to attenuate roxadustat-mediated inhibition of *I*_K(DR)_ or *I*_h_ amplitude. In this scenario, the inhibition by this agent of ionic currents shown here is, thus, unlikely to be mediated largely through the modifications of K_ATP_-channel activity.

In our study, the addition of CoCl_2_ or DFO to the bath could not modify the *I*_K(DR)_ elicited in response to membrane depolarization in GH_3_ cells. These are hypoxia mimetic regents known to produce the induction of HIF-1α [38]. Moreover, in H9c2 cells preincubated with high glucose (30 mM), the biophysical properties of *I*_K(DR)_ remained unchanged, i.e., outward K^+^ current with little inactivation elicited by sustained depolarization. Furthermore, in these cells, roxadustat was also effective at suppressing *I*_K(DR)_ together with a measurable shortening of inactivation time constant elicited by 10-sec maintained depolarization. The preincubation of H9c2 cells with high-glucose medium was previously reported to suppress HIF prolyl hydroxylase and then to cause the upregulation of HIF [30,48]. Therefore, findings from the present experiments prompted us to propose that the ability of roxadustat to suppress the amplitude of *I*_K(DR)_, *I*_Na_, or both is most likely to occur in a non-genomic manner and that it would not be associated with either the inhibition of HIF prolyl hydroxylase or the elevation in the level of HIF [30].

Following a single oral dose of roxadustat at 0.3, 1, 2, 3 or 4 mg/kg, the mean maximal concentrations of roxadustat were previously reported to be 1, 5, 11, 17 and 26 μg/mL (i.e., 2.8, 14.2, 31.2, 48.2 or 73.8 μM), respectively [1,52,53]. In this study, the IC_50_ values required for roxadustat-mediated inhibition of peak or late *I*_K(DR)_ in GH_3_ cells were estimated to be 5.71 or 1.32 μM, respectively. These values are quite close to those that are either therapeutically achievable or needed for the inhibitory actions on HIF prolyl hydroxylases. Alternatively, a concentration-dependent increase by roxadustat in the rate of current inactivation can account largely for differential effectiveness in the reduction in peak and late *I*_K(DR)_. In other words, the inhibitory action of roxadustat on *I*_K(DR)_ is shown to correlate over time with a conceivable rise in the inactivation rate of the current elicited in response to long-step membrane depolarization. The *I*_K(DR)_ recovery from the block during cell exposure to roxadustat was also demonstrated. According to our experimental results, it might be assumed that the blocking site of roxadustat appears to be located within the K_V_ channel pore, only when the channel is open. As such, apart from its inhibitory action at HIF prolyl hydroxylases, the concentration of roxadustat used for both inhibition of ionic currents and modifications in current kinetics (e.g., *I*_K(DR)_ and *I*_Na_ inactivation, or *I*_h_ activation) shown here is conceivably achievable and, thus, of clinical relevance in humans. Findings from the present results would strengthen the notion that the importance of roxadustat-mediated inhibition of HIF prolyl hydroxylase tends to be overestimated, despite the fact that these enzymes could be present in pituitary or heart cells [54]. Roxadustat or other structural similar compounds could also be a family of valuable tools for probing the structure and function of K_V_ channels from the K_DR_ family, as the pore region of the channel protein to which it binds is of particular relevance for open-channel blockade.

Aconitine, a potent cardiotoxin, was previously reported to modify the amplitude and gating of *I*_K(DR)_ in neurons and heart cells [39,46]. Shenfu injection, the composition of which contains aconitine, has been demonstrated to be beneficial for secondary aplastic anemia induced following chemotherapy via stimulation of bone marrow function [6,55,56]. Previous studies have demonstrated the ability of aconitine to depress *I*_K(DR)_ amplitude and to fasten the inactivation rate of the current [39,46]. Therefore, because of their similar actions on the amplitude and gating of *I*_K(DR)_, to what extent changes in the amplitude and gating of *I*_K(DR)_ by aconitine or roxadustat could produce any stimulatory effects on the function of hematopoietic cells in bone marrow [57] is worthy of being further investigated. Additionally, it needs to be noted that in *Kcnk3*-mutated rats, a novel model for occurrence of pulmonary hypertension was recently characterized [58].

There are limitations in the present study. Ideally, it would be important to evaluate the effects of roxadustat on ionic currents, when the mRNAs of hypoxia inducible factor-1α (HIF-1α) have been knocked down, and subsequently to test the perturbations on all the ion channels of interest in these cells. However, under our experimental results, the rapid onset of actions of roxadustat on ionic currents seen in GH_3_ or H9c2 cells were noted. Moreover, the cells in which the HIF-1α mRNAs were knocked-down, would have become too fragile to be accurately studied during the measurements. For example, the activity of ATP-sensitive K^+^ channels in GH_3_ or H9c2 cells would have been seriously deranged [27,49,50,54]. Alternatively, in our study, in high-glucose-treated H9c2 cardiac cells, inhibition by roxadustat of the *I*_K(DR)_ elicited in response to long-lasing membrane depolarization was noted to remain efficacious. It needs to be mentioned that the challenging of cells with high glucose has been demonstrated to suppress the activity of HIF prolyl hydroxylases along with the upregulation of HIF [30,47,48] We also found out that deferoxamine, an inhibitor of iron-dependent prolyl hydroxylase [38], could not modify the amplitude or gating of *I*_K(DR)_ in GH_3_ cells. The results thus reflect the fact that roxadustat-mediated inhibition of *I*_K(DR)_ in GH_3_ cells tends to be not attributable to its actions on the accumulation of HIF-1α. To what extent roxadustat-mediated modifications of ionic currents can be potentially altered in HIF-1α-knocked down cells remains to be further delineated.

Nonetheless, our studies indeed highlight the notion that, in addition to the inhibition of HIF prolyl hydroxylase, the roxadustat molecule per se is capable of interacting directly with membrane ion channels, thus perturbing the amplitude and gating of ionic currents. Therefore, roxadustat is recognized as a potent and efficacious compound in suppressing ionic currents (e.g., *I*_K(DR)_) in a non-genomic pathway. The interaction of roxadustat with the activity of these ion channels presented herein is acute in onset and thus less likely to be linked to the activity of HIF-1α, although the detailed molecular mechanism of its action on ionic currents still needs to be further resolved.

## 4. Materials and Methods 

### 4.1. Drugs, Chemicals and Solutions

Roxadustat (FG-4592, ASP1517, AZD9941, Ai Rui Zhuo^®^, Beijing, China, N-[(4-hydroxy-1-methyl-7-phenoxy-3-isoquinolinyl)carbonyl]-glycine, C_19_H_16_N_2_O_5_, https://pubchem.ncbi.nlm.nih.gov/compound/Roxadustat) was acquired from Cayman (Excel Biomedical Inc., Taipei, Taiwan), diazoxide, nonactin and PD-118057 were from Tocris (Union Biomed Inc., Taipei, Taiwan), and deferoxamine (DFO), oxaliplatin and tetrodotoxin were from Sigma-Aldrich (Merck Ltd., Taipei, Taiwan). Tissue culture media, fetal calf serum, horse serum, L-glutamine, penicillin-streptomycin, fungizone and trypsin/EDTA were obtained from Invitrogen Corp. (Carlsbad, CA, USA), and other chemicals that include CdCl_2_, CoCl_2_, CsCl, CsOH, aspartic acid and HEPES (4-(2-hydroxyethyl)-1-piperazineethanesulfonic acid) were of the best available quality, mostly at analytical grades.

The composition of normal Tyrode’s solution was 136.5 mM NaCl, 5.4 mM KCl, 1.8 mM CaCl_2_, 0.53 mM MgCl_2_, 5.5 mM glucose, and 5.5 mM HEPES-NaOH buffer, pH 7.4. To record *I*_K(DR)_ or *I*_h_, the patch electrode was filled with the following solution: 140 mM KCl, 1 mM MgCl_2_, 3 mM Na_2_ATP, 0.1 mM Na_2_GTP, 0.1 mM EGTA, and 5 mM HEPES-KOH buffer, pH 7.2. To measure *I*_Na_, we replaced KCl inside the pipette solution with equimolar CsCl, and pH was adjusted to 7.2 with CsOH. To avoid possible contamination of Cl^-^ currents, we substituted aspartate for Cl^-^ ions inside the pipette solution. To measure *I*_K(erg)_, bath solution was replaced with a high-K^+^, Ca^2+^-free solution containing 130 mM KCl, 10 mM NaCl, 3 mM MgCl_2_, and 5 mM HEPES-KOH buffer, pH 7.4, and the pipette solution contained 145 mM KCl, 2 mM MgCl_2_, and 5 mM HEPES-KOH buffer, pH 7.2. 

### 4.2. Cell Culture

GH_3_ pituitary tumor cells, obtained from the Bioresource Collection and Research Center ([BCRC-60015]; Hsinchu, Taiwan), were grown in Ham’s F-12 medium supplemented with 15% horse serum (*v*/*v*), 2.5% fetal bovine serum (*v/v*), and 2 mM L-glutamine [59]. To promote cell differentiation, GH_3_ cells were transferred to a serum-free, Ca^2+^-free medium. The H9c2 cell line, derived from embryonic rat ventricles, was also provided by the Bioresource Collection and Research Center (BCRC-60096). Cells were grown in Dulbecco’s modified Eagle’s medium supplemented with 10% fetal bovine serum (*v*/*v*) and 2 mM glutamine [45]. GH_3_ or H9c2 cells were maintained at 37 °C in a humidified environment of 5% CO_2_/95% air and sub-cultured weekly and fresh media were replenished every 2–3 days for removal of non-adhering cells. In another separate set of experiments, in attempts to suppress the activity of HIF prolyl hydroxylase [30], we incubated H9c2 cells in high-glucose medium containing 30 mM glucose and 2% fetal bovine serum for 24 h.

### 4.3. Electrophysiological Measurements

On the day of each experiment, GH_3_ or H9c2 cells were dissociated and an aliquot of cell suspension was transferred to a custom-built recording chamber firmly mounted on the stage of an inverted DM-IL microscope (Leica, Wetzlar, Germany). Cells were bathed at room temperature (20–25 °C) in normal Tyrode’s solution, the composition of which is described above. The pipettes were fabricated from Kimax-51 borosilicate capillaries (#34500; Kimble, Vineland, NJ, USA) by using either a PP-83 vertical puller (Narishige, Tokyo, Japan) or a P-97 Flaming/Brown horizontal puller (Sutter, Novato, CA, USA). The electrodes used for the recordings had a tip resistance ranging between 3 and 5 MΩ, when they were filled up with different internal solutions described above. Ion currents were measured in the whole-cell mode of standard patch-clamp technique by use of either an RK-400 (Bio-Logic, Claix, France) or an Axopatch 200B (Molecular Devices, Sunnyvale, CA, USA) patch amplifier [41,59]. All potentials were corrected for liquid–liquid junction potential that would appear, when the composition of the pipette solution remained different from that in the bath. Through digital-to-analog conversion, the voltage-step profiles including one- or two-step pulses digitally, which were created from pCLAMP 10.7 software (Molecular Devices), were particularly designed to establish either the current–voltage (*I–V*) relations of ionic currents, or the steady-state inactivation and recovery from inactivation of *I*_K(DR)_.

### 4.4. Data Recordings

The signals, consisting of potential and current traces, were stored online at 10 kHz in an ASUSPRO-BU401LG computer (ASUS, Taipei City, Taiwan) through a Digidata 1440A interface (Molecular Devices, Sunnyvale, CA, USA), the device of which was controlled by pCLAMP 10.7 (Molecular Devices, Sunnyvale, CA, USA). The laptop computer was put on the top of an adjustable Cookskin stand (Ningbo, Zhejiang, China) for efficient control during the recordings. Current signals were low-pass filtered at 3 kHz. The data digitally acquired during each experiment were analyzed subsequently using different analytical tools that include LabChart 7.0 program (AD Instruments; Gerin, Tainan, Taiwan), OriginPro (Microcal, Northampton, MA, USA), and custom-made macro procedures built under Microsoft Excel^TM^ 2016 (Redmond, WA, USA).

### 4.5. Data Analyses

To evaluate concentration-dependent inhibition of roxadustat on the amplitude of *I*_K(DR)_, cells were bathed in Ca^2+^-free Tyrode’s solution and step depolarizing pulses from −50 to +50 mV with a duration of 1 sec were delivered. The *I*_K(DR)_ amplitude which was measured at the beginning (i.e., peak component) or end (i.e., late component) of each long-step depolarizing pulse, during cell exposure to different concentrations (0.3–100 μM) of roxadustat was compared with the control value. The concentration-dependent relation of roxadustat on the inhibition of *I*_K(DR)_ measured in GH_3_ cells was established by fitting the data with the goodness of fit to a modified form of the Hill equation. That is, where [Roxa], IC_50_, and n_H_ are the roxadustat concentration used, the concentration required for a 50% inhibition, and Hill coefficient, respectively; and, A or a represents the maximal fraction of total current (peak and late component) which is roxadustat sensitive or insensitive, respectively.

(1) $$\frac{I_{\mathrm{Roxa}}}{I_{\mathrm{control}}}=A\times \left(\frac{{\left[\mathrm{Roxa}\right]}^{n_{H}}}{{\mathrm{IC}}_{50}^{n_{H}}+{\left[\mathrm{Roxa}\right]}^{n_{H}}}\right)+a$$

To determine the steady-state inactivation curve of *I*_K(DR)_ during cell exposure to different roxadustat concentrations, the relationships between the normalized amplitude of *I*_K(DR)_ and the conditioning potentials obtained in the presence of 3 or 10 μM roxadustat were least-squares fitted with a modified Boltzmann function of the following form:

(2) $$\frac{I}{I_{\max}}=\left(\frac{1-a}{1+\exp\left[(V-V_{\frac{1}{2}})\mathrm{qF}/\mathrm{RT}\right]}\right)+a$$

where *I*_max_ is the maximal peak amplitude of *I*_K(DR)_ taken at the level of +50 mV, *V*_1/2_ the voltage at which half-maximal inhibition occurs, *q* the apparent gating charge of the inactivation curve, *F* Faraday’s constant, *R* the universal gas constant, *T* the absolute temperature, and *a* the residual fraction of the current.

### 4.6. Statistical Analyses

Linear or non-linear curve-fitting (e.g., single or two exponential, or sigmoidal fitting) to data sets was performed with the goodness of fit by using different maneuvers such as Microsoft Solver function embedded in Excel 2016 (Microsoft) and 64-bit OriginPro 2016 program (OriginLab). The averaged results are presented as the mean±standard error of the mean (SEM) with sample sizes (*n*) indicating the cell numbers from which the results were collected. The paired or unpaired Student’s *t*-test and a one-way analysis of variance (ANOVA) followed by post hoc Fisher’s least-significance difference method, were implemented for statistical evaluation of differences among means. If normality underlying ANOVA was presumably violated, we attempted to use the non-parametric Kruskal–Wallis test. Statistical analyses were performed using the SPSS 20 statistical software package (IBM Corp., Armonk, New York, USA). Statistical significance was determined at a *p*-value of <0.05.

## 5. Conclusions

In the present study, we found that roxadustat was effective at modifying the amplitude and gating of *I*_K(DR)_ in GH_3_ and H9c2 cells. The modification of aconitine on *I*_K(DR)_ was reported to aberrantly prolong action potential in heart cells, in combination with the emergence of polymorphic ventricular tachycardia. Therefore, whether roxadustat caused any serious complications in heart function needs to be determined as an imperative, despite a previous study showing little or no occurrence of serious adverse cardiac events during roxadustat treatment. Nonetheless, our study reveals another unidentified but important aspect which needs to be given due consideration, despite the inhibitory actions of either roxadustat or other structurally similar compounds on the activity of HIF prolyl hydroxylase inside the cell. Such actions on these ionic currents are of particular clinical, pharmacological or toxicological relevance.

## Abbreviations

DFO	Deferoxamine
*erg*	*ether-à-go-go*-related gene
HIF	Hypoxia-inducible factor
IC_50_	The concentration required for 50% inhibition
*I*_h_	Hyperpolarization-activated cation current
*I*_K(DR)_	Delayed-rectifier K^+^ current
*I*_K(erg)_	*erg*-mediated K^+^ current
*I*_Na_	Voltage-gated Na^+^ current
K_ATP_ channel	ATP-sensitive K^+^ channel
roxadustat	(FG-4592, N-[(4-hydroxy-1-methyl-7-phenoxy-3-isoquinolinyl)carbonyl]-glycine)
SEM	Standard error of the mean
τ_inact(F)_	Fast component of inactivation time constant
τ_inact(S)_	Slow component of inactivation time constant
